# Editorial: Immune functions of neuroglia

**DOI:** 10.3389/fimmu.2026.1885437

**Published:** 2026-06-16

**Authors:** Friederike Pfeiffer, Alexei Verkhratsky, Olga Garaschuk

**Affiliations:** 1 Institute of Physiology, Department of Neurophysiology, Eberhard Karls University of Tübingen, Tübingen, Germany; 2 Faculty of Biology, Medicine and Health, The University of Manchester, Manchester, United Kingdom

**Keywords:** astrocyte, inflammation, macrophage, microglia, neurodegenerative diseases, oligodendroccyte, phagocytosis, T cell

**Figure 1:**
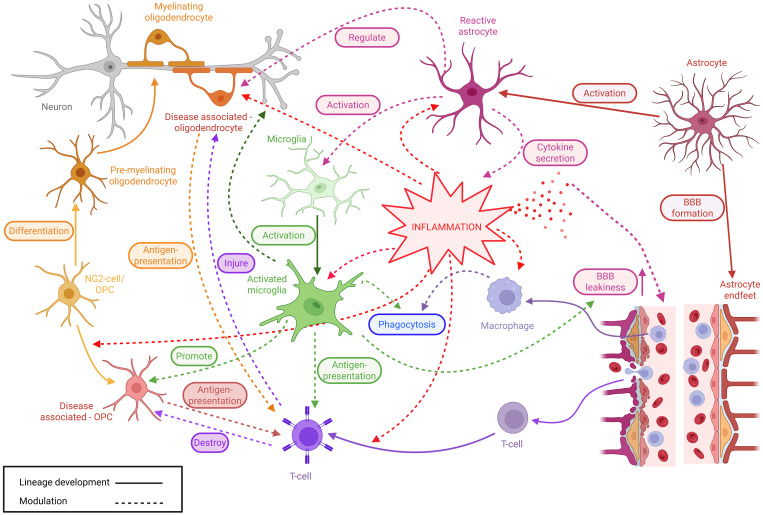
Reactive/disease-associated glial cells increase under inflammatory conditions and contribute to neuroinflammation. Created in BioRender. Pfeiffer, F. (2026) https://BioRender.com/qg0l68t. .

Neuroglial cells are pivotal regulators of innate and adaptive immune processes within the brain and spinal cord. Neuroglia, comprising microglia, astrocytes, oligodendrocytes, oligodendrocyte precursor cells (OPCs), and ependymal cells orchestrate a complex network of immune surveillance, signaling, and blood–brain barrier maintenance, thereby controlling immune cell entry (**Figure 1**). Microglia function as the resident macrophage-like cells of the central nervous system (CNS) with numerous physiological functions. Microglia continuously monitor the neural environment and rapidly respond to injury, infection, and perturbations in homeostasis, with changes in process motility, polarization, directed process movement, morphology, and gene expression profiles. Microglia are also fundamental to the regulation of neural cell proliferation, phagocytosis, synaptic pruning and the release of signaling molecules or pro- and anti-inflammatory mediators (1, 2).

Astrocytes strongly contribute to CNS immune responses. Through endfeet, they contribute to the formation and maintenance of the blood–brain barrier (BBB) and perivascular space, thereby controlling immune cell entry. Astrocytes respond to injury or infection through a process termed reactive astrogliosis (3), characterized by morphological and transcriptional changes that can either limit damage or contribute to chronic inflammation (**Figure 1**). In both physiological and pathological contexts, astrocytes secrete cytokines, chemokines, and growth factors controlling reactive microgliosis and peripheral immune cell recruitment. Astrocytes also regulate extracellular ion balance, neurotransmitter clearance, and metabolic support, all of which indirectly shape inflammatory signaling (4). Oligodendrocytes and OPCs (**Figure 1**), while primarily involved in myelination, also participate in immune interactions and synaptic pruning and are particularly vulnerable to immune-mediated damage. Finally, ependymal glia control CNS-cerebrospinal fluid (CSF) communications involved in the regulation of CNS immunity. Together, these glial populations form an integrated immunological framework essential for maintaining homeostasis of the nervous tissue and coordinating protective responses. When dysregulated, they contribute to the pathogenesis of neuroinflammatory and neurodegenerative disorders.

This research topic aims to explore the potential immune functions of different glial cell types and their interactions with peripheral immune cells that modulate immune responses, and to address the underlying mechanisms, which are often related but not limited to macroglia-microglia interactions.

The review by Haroon et al. provides an overview of what is known about the recently recognized immune capabilities of OPCs, including phagocytosis of myelin debris and the engulfment of axons and presynaptic terminals via low-density lipoprotein receptor-related protein 1 (LRP1). In addition, the authors discuss OPCs’ ability to release specific cytokines that regulate the activation and recruitment of immune cells (**Figure 1**). A subset of OPCs can even present antigen to T cells by expressing major histocompatibility complexes, contributing to the pathogenesis of Multiple Sclerosis (MS). Thus, the functions of OPCs are far more complex than we have anticipated thus far. Similar to what has been described for microglia, oligodendrocytes and OPCs may also adopt disease-associated phenotypes that differ from those that contribute to myelin formation under homeostatic conditions (5).

MS usually starts with a relapsing-remitting form that then later converts to a progressive form with age and duration of the disease. Atkinson et al. summarize the contribution of microglia to CNS infiltration by T cells, driving the progressive phase of MS and its animal models during aging. The latter specifically target oligodendroglial cells and impair their potential to myelinate. The authors present evidence for the beneficial effects of senolytic therapies on clinical scores in a mouse model of MS, using middle-aged rather than young mice, highlighting the importance of individual patients’ age for their vulnerability to disease progression and their responsiveness to therapies. Thus, the interplay between microglia and T cells can modulate the microenvironment of OPCs and thereby influence their remyelination efficiency.

Uzcategui et al. describe, in a mouse model, microglia response to CNS invasion by the parasite *Trypanosoma brucei*. They show that the parasite load increases in the CSF but not in the brain parenchyma, despite obvious early reactive microgliosis. These findings highlight the important role of microglia in brain protection and their capacity to fight parasitic infection together with infiltrating peripheral macrophages. This study also illustrates the interplay between resident CNS immune cells (microglia) and peripheral immune cells in the CSF, elucidating the sequential involvement of these cells in an *in vivo* model.

Along these lines, Tan et al. delve into the distinct roles that microglia and monocyte-derived macrophages (MDMs) play in defending the retina against inflammation. While microglial cells are responsible for immune surveillance, synaptic pruning, debris clearance, regulation of vascular development and secretion of neurotrophic factors in the healthy retina, the appearance of MDMs in the retina, similar to what was described above for an inflammatory setting at the meninges and in the brain parenchyma, is a sign of disruption of the blood-retina barrier and homeostatic imbalance. This review also discusses the possibilities for distinguishing the two phagocytic cell populations once they reside within the retina at different stages of glaucoma, as well as their distinct activation states and functions, an important prerequisite for specific targeting.

In addition to the protective role of neuroglia in combating CNS infections, inflammatory reactions can have detrimental effects on brain health. In an *in vitro* model of neurodegeneration, Reid and Brown showed that microglia secrete low-density lipoprotein receptor-related protein-associated protein 1 (LRPAP1), most likely in an attempt to protect neurons from the removal of synapses by phagocytosis, which subsequently inhibits the uptake of amyloid β, thus preventing its clearance and export from the brain via LRP1. Interestingly, OPCs use the same phagocytic receptor, LRP1, to internalize synapses (6), and LRP1 is used by glial cells and macrophages to clear myelin debris (7).

In summary, much remains to be learned about the immune functions of neuroglia in the CNS, with great potential for developing new therapies. And, it is becoming increasingly clear that it is not enough to focus on them independently. One must consider their interactions and mutual influence. In the context of brain diseases that lead to BBB breakdown (e.g., inflammation and neurodegeneration), the contributions of peripheral immune cells and modulation of their functional properties by local glial cells must also be considered.
